# Differences in well-being and fear of death among female hospice employees and volunteers in Hungary

**DOI:** 10.1186/s12904-020-00550-z

**Published:** 2020-04-24

**Authors:** Ágnes Zana, Adrienne Kegye, Edit Czeglédi, Katalin Hegedűs

**Affiliations:** Faculty of Medicine, Institute of Behavioural Sciences, Semmelweis University, 1089 Budapest, Nagyvárad tér 4, Budapest, Hungary

**Keywords:** Hospice, Volunteer, Fear of death, Mental health

## Abstract

**Background:**

Voluntary work plays a significant role in hospice care, but international research has mainly been conducted on the mental health and fear of death of paid hospice staff. The aim of the present study was to compare the Hungarian hospice volunteers with paid employees with regard to attitudes and fear of death, as well as mental health in order to see their role in hospice work and their psychological well-being more clearly.

**Methods:**

The target population of the cross-sectional questionnaire study was hospice care providers in Hungary (*N* = 1255). The response rate was 15.5% (*N* = 195); 91.8% (*N* = 179) of them were women. The mean age of female hospice workers was 45.8 years (SD = 10.46 years, range: 23–73 years). One-quarter (27.9%, *N* = 50) of the female respondents were volunteers. The instruments were: the Multidimensional Fear of Death Scale, the Perceived Stress Scale, the WHO-5 Well-Being Index, and a shortened versions of the Beck Depression Inventory and the Maastricht Vital Exhaustion Questionnaire.

**Results:**

Volunteers scored significantly lower on 5 dimensions of fear of death than paid employees, and showed significantly lower levels of vital exhaustion and significantly higher levels of psychological well-being than paid employees. Fear of the dying process was associated with an increased perceived stress, depressive symptoms, and vital exhaustion in both groups. Psychological well-being showed a significant negative, moderate correlation with four aspects of fear of death among paid staff; this pattern did not appear in the volunteer group. In addition, the association between fear of premature death and perceived stress, vital exhaustion, and depressive symptoms was more pronounced is case of paid workers.

**Conclusion:**

Higher levels of psychological well-being and lower levels of fear of death among hospice volunteers suggest that they are less exhausted than paid employees. Increasing the recruitment of volunteers in hospices may help reduce the overload and exhaustion of paid employees.

## Background

Unlike well-trained staff, volunteers undertake this highly challenging work at hospice and palliative care for free, but so far, research has been less focused on their psychological well-being and relationship to death, which are important factors in shaping their place in the team. The presence of voluntary helpers is essential in end-of-life care. According to a study by Block et al. [1] a higher number of volunteers employed in care was directly proportional to a higher rating given by families to quality of care. Thus, satisfaction with care services can be improved by adequately qualified volunteers who are psychologically prepared for the work. Volunteers perform mentally demanding jobs by working with dying people without any financial compensation. It is important to preserve volunteers’ mental health. In accordance with this objective, in Hungary it is obligatory for every healthcare worker and volunteer to complete a 40-h basic training course, 20 h of which are based on personal experience, dealing with death, personal reflection, and awareness related to loss. During the obligatory 40-h training, we assess their emotional capacity in order to select only those who are psychologically fit to take part in bedside care and who do not have to deal with loss in their current life circumstances [2–4].

In international hospice and palliative care, in line with the improvement of volunteering, the number of studies investigating volunteering has increased in recent years (searching for keywords “volunteer” and “hospice” in PubMed results in 153 hits between years 2000–2009, 274 hits between years 2010–2019). In the latter period, the quality of volunteering changed. The literature shows that a different kind of volunteering was typical in the 1980s: earlier volunteering was related to traditional civic values, solidarity, family-based motivation, and ultimately to religious background, while the “new” kind of volunteering is instead an activity that aims at knowledge acquisition, meaningful use of free time, and the improvement of self-awareness [5]. A study conducted with 125 volunteers in American and German cultural groups showed that the old and new kind of volunteering both appeared as altruism in the two samples. The same study compared hospice volunteers to other (non-healthcare) volunteers, and found significant differences. Altruism was a significant factor for hospice volunteers of both cultures, but German non-hospice volunteers were motivated by self-improvement and learning opportunities as well [6].

The growing number of volunteers prompted the European Association for Palliative Care (EAPC) to issue recommendations for the development of higher quality hospice volunteering that is more coherent and sustainable. The EAPC presented the Volunteering Charter (“Voice of Volunteering” - The EAPC Madrid Charter on Volunteering in Hospice and Palliative Care, 2017, Madrid, Spain) during the 2017 World Congress in Madrid. The charter contains recommendations and short-term goals concerning the improvement of hospice volunteering. Representatives of countries working together as working groups are to elaborate, formulate and establish the main guidelines disseminated at the Congress.

The EAPC volunteering - work group is utilizing a country-by-country comparative study of European volunteering and its possible improvement. Seven countries are involved in the study, mainly from Western Europe [7]; there were 340 registered volunteers in Belgium, 70,000 in England, and 85,000 in Germany. When pondering these numbers, the differences in the size of population must be taken into account, as well as various levels of experience in self-organization, deriving from different traditions in hospice care. A summative study volume published in 2013 [8] discusses the experience of hospice volunteers in the United States. The cross-sectional study showed that adequate training significantly contributed to the efficiency of volunteers and to their retention in the system.

Hungarian data is most aptly compared to Belgian data, considering the size of the population. In Hungary, data from 2016 shows that out of 1255 hospice workers, 208 are volunteers (165 of whom are contractual volunteers) working in different services [9]. The international study shows that volunteers are deployed in a variety of fields and receive different trainings [7]. In Hungary, the recruitment and training of volunteers is not performed by one institution, but rather, several independent hospice services assume this task – unlike, for example, the Polish Caritas [10].

One of the goals of the international initiative is to help develop hospice volunteering on a national level. As experience is shared on an international scale, it is evident that issues of volunteer recruitment, training and employment are significantly different due to cultural and socio-economic reasons. Yet, the common goal is to achieve a more uniform and higher level of training. The prerequisite for effective training is the mapping of volunteers’ mental health beyond their socio-cultural conditions [11]. Therefore, we will review the literature on factors below.

The mental status and socio-cultural situation are related to attitudes towards death and fear of death, according to previous research [11]. Research so far has primarily concentrated on paid employees’ attitude towards death compared with their mental state [12, 13]; volunteers have only recently come entered the focus of attention.

Amenta [14] was the first to measure fear of death among hospice volunteers. The author employed the Maholick Purpose in Life Test and the Templer Death Anxiety Scale [15]; 42 predominantly female hospice volunteers submitted the questionnaire; the mean age was 40 years. Amenta [14] found that the more life goals one has, the less they are afraid of death. Although the definition of death anxiety and fear of death have varying interpretations, a noteworthy finding in the study is that there is a need for the measurement of volunteers’ anxiety/fear. Fear of death consists of more specific, definable, conscious factors, while the anxiety of death is a vague, indescribable fear [16]. The *Death Anxiety Handbook* was published in 1994, the corrected version of the Multidimensional Fear of Death Scale (MFODS) was first published here [17]. The book addresses hospice volunteers’ attitudes towards death and examines the effects of death-related trainings [18]. According to this, trainings significantly reduced anxiety related to death, had a positive influence on the participants’ attitudes towards death, and improved their faith in the meaningfulness of life.

In a French study [19] correlations between volunteers’ motivation and their fear/anxiety related to death were investigated (*N* = 113). It was found that death anxiety is lower for hospice volunteers and that motivation is primarily altruistic, compared with those of French non-hospice volunteers and non-volunteers.

A review examining a span of 10 years found that regarding stressors, volunteers highlighted communication difficulties, lack of emotional support, training and informational needs, taking care of the patient’s family members, and coping with death and the dying process [7].

From 2000 onwards, the main purpose of studies on the attitude of volunteers has been to increase their work efficiency. Most research findings are from the United States, Canada, and the United Kingdom because these are the countries with the strongest volunteering traditions. An American study (*N* = 46) revealed that among those volunteers who also learned yoga, death anxiety was much lower [20]. Another American study applied the Collet-Lester Fear of Death Scale [21]. In this study, 17 participants answered the questionnaires before and after the training course; the purpose of the research was to measure training efficiency. Findings of the research show that volunteers’ attitudes towards death improved, but levels of fear of death did not change significantly.

Work efficiency of hospice staff can be improved by decreasing fear of death because confronting and managing your own fear of death facilitates communication with incurable patients. Trainings for special subgroups play a vital role in this process. All of this can contribute to optimizing hospice specialist training on the one hand, and developing psychological support for hospice and palliative care providers on the other.

### Research objectives

Our goals were to explore the dimension of fear of death among hospice workers and paid staff in Hungary, to compare fear of death and mental health of volunteer workers with paid employees, and to examine psychological correlates (such as perceived stress, depression, vital exhaustion and well-being) of fear of death.

Based on our previous research, we assumed that volunteers have a higher fear of death than paid workers [22].

## Methods

### Participants and procedure

Hungarian hospice care staff (*N* = 1255) were involved in the cross-sectional questionnaire survey (Ethics certificate number: 274/2013, TUKEB). The participants gave written informed consent for participation in the study. The questionnaires were available online and in paper-based form; 195 valid questionnaires were received. The response rate was 15.5, and 91.8% (*N* = 179) of the respondents were women. Due to the low number of males (*N* = 16), analysis was carried out only on the females in the sample.

71.5% (*N* = 128) of the respondents were paid staff. Most of them are nurses (44.1%), the remaining 56% are other team members: doctors, social workers, psychologists, physiotherapists, priests, dieticians. Volunteers comprised 27.9% (*N* = 50) of the sample and there was one missing data (0,6%) in this regard.

The mean age of female participants was 45.8 years (SD = 10.46 years, range: 23–73 years). Regarding educational qualifications, 2.8% (*N* = 5) of the sample had completed primary education (8 classes of primary school and basic professional qualifications), 34.1% (*N* = 61) had completed secondary education (graduation examination and professional qualifications), and 63.1% (*N* = 113) had obtained a higher education degree. 33.5% of the respondents (*N* = 60) lived alone, while 66.5% (*N* = 119) lived with a partner. With respect to age, we found that volunteers are significantly older than paid staff (M = 49.1 (SD = 12.30) years vs. M = 44.3 (SD = 9.26) years, t (71.74) = 2.508, *p* = 0.014). After combining respondents with primary and secondary education chi-square test did not show a significant difference between volunteers and paid employees in terms of highest educational attainment (χ^2^(1) = 1.274, *p* = 0.259). However, paid employees were significantly more likely to live with partners than volunteers (71.1% vs. 56.0%, (χ^2^(1) = 3.697, *p* = 0.055).

### Instruments

The Hungarian validated version of the following questionnaires were employed in the study:

1. The Multidimensional Fear of Death Scale (MFODS) [23] consists of 42 items categorized into eight factors. The factors are the following: *Fear of the Dying Process* (including painful and violent deaths); *Fear of the Dead* (including avoidance of both human and animal remains); *Fear of Being Destroyed* (including dissection and cremation of the body); *Fear for Significant Others* (including both apprehension about the death of persons important to us and their apprehension about our death); *Fear of the Unknown* (fear of the cessation of being and of life after death or its absence); *Fear of Conscious Death* (anxieties about falsely being declared dead while still alive); *Fear for the Body After Death* (concerns about decay and isolation of the body); and *Fear of Premature Death* (concerns that death will prevent us from accomplishing important life goals or having significant experiences). Items are rated on a five-point Likert scale ranging from strongly disagree (1) to strongly agree (5). Higher scores on scales indicate greater fear.^1^ The internal consistency of the Hungarian version of the questionnaire, except for the Fear of Death Consciousness Scale (Cronbach’s alpha = 0.45), is acceptable (Cronbach’s alpha: 0.58–0.76) [24].
2. The Perceived Stress Scale – 10 Items [25] is a ten-item questionnaire that assesses the presence of thoughts and feelings associated with perceived stress, the responder’s subjective stress experiences in the past month, as well as perceived everyday unpredictability, loss of control, and overburdening. Items are rated on a five-point Likert scale ranging from never (0) to very often (4). Higher scores indicate a higher level of perceived stress. The internal consistency of the Hungarian version was adequate (Cronbach’s alpha: 0.85) [26].
3. The WHO (Five) Well-Being Index [27] is a five-item scale that assesses the general well-being of the respondents (including good mood, calmness, and relaxedness) in the past 2 weeks. Items are rated on a four-point Likert scale ranging from very atypical (0) to very typical (3). Higher scores on this scale indicate a higher level of psychological well-being. The reliability of the scale was adequate in a representative Hungarian sample (Cronbach’s alpha: 0.85) [28].
4. The Shortened Beck Depression Inventory is a nine-item questionnaire and a reliable tool for the measurement of depression symptom severity [29]. The items are rated on a four-point Likert scale, possible responses ranged from very atypical (1) to very typical (4). Higher scores indicate a higher level of depressive symptoms. The internal consistency of the scale was adequate in a representative Hungarian sample (Cronbach’s alpha: 0.92) [30].
5. The Shortened Maastricht Vital Exhaustion Questionnaire [31], which is a five-item dichotomous instrument (possible responses: yes or no) that measures the experience of vital exhaustion, consisting of tiredness, a lack of energy, and increased irritability. Higher scores indicate a greater degree of vital exhaustion. The internal consistency of the scale was adequate in a representative Hungarian sample (Cronbach’s alpha: 0.78) [28].

### Statistical analyses

Cronbach’s alpha was used for the assessment of the internal consistency of the scales. In case of continuous variables, voluntary and paid hospice staff groups were compared via independent samples t-test or the Mann-Whitney U-test, while in the case of categorical variables, we used the chi-square test for comparison. We applied correlation analysis to explore the linear relationships between continuous variables (Spearman correlation methods). For the interpretation of the correlation coefficients, we took Cohen’s definition [32] as a basis, i.e. the effect size is small under 0.3, medium between 0.3 and 0.5, and large above 0.5.

## Results

### Descriptives

Table 1 shows the descriptive statistics of the investigation and the comparison of volunteer and paid staff regarding all variables. Internal consistency of the questionnaires proved to be sufficient during this survey, except for three MFODS scales. For the scales *Fear of the Dead, Fear of the Unknown,* and *Fear for the Body after Death*, reverse items always decreased internal reliability significantly. Therefore, analyses have been carried out with reverse items excluded from the scales.

**Table 1:** Basic descriptive statistics and comparison of volunteers and paid staff regarding specific variables

Variable	Cronbach-alpha (item number)	Total sample (*N* = 168–176)	Volunteers (*N* = 44–49)	Paid staff (*N* = 123–127)	Z/t (p)
		Mean (SD)			
Fear of the dying process	0,77 (6)	15.4 (5.55)	13.6 (5.19)	16.0 (5.55)	Z = 2.392* (*p* **= 0.017)**
Fear of the dead^a^	0.60 (5)	10.2 (3.79)	9.2 (3.50)	10.5 (3.84)	Z = 2.029* (*p* **= 0.042)**
Fear of being destroyed	0.60 (4)	9.8 (4.06)	8.9 (4.11)	10.1 (4.00)	Z = 1.856^+^ (*p* = 0.063)
Fear for significant others	0.71 (6)	20.7 (4.72)	19.0 (4.86)	21.3 (4.54)	t (170) = −2.898** (*p* **= 0.004)**
Fear of the unknown^a^	0.67 (4)	8.0 (3.53)	7.8 (3.91)	8.0 (3.41)	Z = 0.555 (0.579)
Fear of conscious death	0.68 (5)	7.7 (3.48)	7.5 (3.70)	7.7 (3.40)	Z = 0.337 (*p* = 0.736)
Fear of the body after death	0.72 (5)	10.2 (3.60)	9.4 (3.65)	10.5 (3.55)	Z = 2.225* (*p* **= 0.026)**
Fear of premature death	0.82 (4)	8.7 (4.16)	7.4 (4.19)	9.2 (4.06)	Z = 2.993** (*p* **= 0.003)**
Shortened Beck’s Depression Inventory (BDI)	0.86 (9)	2.9 (3.76)	2.5 (3.97)	3.0 (3.68)	Z = 1.219 (*p* = 0.223)
Vital exhaustion scale	0.71 (5)	2.2 (1.64)	1.8 (1.55)	2.4 (1.65)	Z = 2.220* (*p* **= 0.026)**
Perceived stress scale	0.88 (10)	26.6 (6.38)	26.0 (7.15)	26.8 (6.09)	t (167) = −0.785 (*p* = 0.434)
WHO well-being scale	0.81 (5)	8.9 (2.74)	9.6 (2.47)	8.6 (2.80)	t (174) = 2.071* (*p* **= 0.040)**

Remark ^a^: in order to improve internal consistency these scales excluded reverse items; they are shorter than the original scales by one item. ^+^*p* < 0.10, * *p* < 0.05, ** *p* < 0.01

Concerning death-related fears, our findings show that the mean of *Fear of the dying process, Fear of the dead, Fear of premature death, Fear for significant others* and *Fear for the body after death* were significantly lower for volunteer workers than those of the paid staff. Furthermore, levels of vital exhaustion were significantly lower, while psychological well-being was significantly higher among volunteers than those of the paid staff.

### Correlates of fear of death

The association between death-related fears and psychological well-being was tested using Spearman’s rank correlation analysis in a sample of volunteers and paid employees. All in all, there were relatively few significant relationships, and these were also primarily found in paid employees. The strength of the relationships was in the weak and moderate range, which dominated the sample of paid employees. It is worth emphasizing that the fear of the dying process shows a significant, positive, moderate correlation with perceived stress, vital exhaustion, and depressive symptoms in both groups. Psychological well-being showed a significant, negative, moderate association with four aspects of fear of death among paid employees; this pattern did not appear in the volunteer group. Correlations of fear of premature death were also more prominent among paid employees in terms of perceived stress, vital exhaustion, and depressive symptoms. Finally, age also showed a significant, negative, weak association between fear of the dead and the fear of significant peers only in the paid employees group. Table 2 shows these results.

**Table 2:** Correlations of fears about death

Correlations	Perceived stress total scores		Vital exhaustion total scores		BDI total scores		WHO well-being total scores		Age	
	Volunteer	Paid staff	Volunteer	Paid staff	Volunteer	Paid staff	Volunteer	Paid staff	Volunteer	Paid staff
Fear of the dying process	0.34*	0.41***	0.36*	0.35***	0.30*	0.35***	**− 0.24**^**+**^	− 0.38***	0.07	− 0.00
Fear of the dead	0.14	0.13	0.17	0.08	0.04	0.08	−0.03	− 0.12	− 0.17	− 0.25**
Fear of being destroyed	0.11	0.01	0.20	−0.01	0.17	0.05	−0.12	−0.06	− 0.01	−0.14
Fear for significant others	0.27^+^	0.03	0.37*	**−0.03**	0.11	−0.03	−0.18	− 0.18*	−0.15	− 0.26**
Fear of the unknown	−0.00	0.04	−0.04	0.07	−0.18	0.11	0.09	−0.10	0.01	−0.13
Fear of conscious death	0.15	0.18*	0.13	0.22*	0.27^+^	0.23*	**−0.03**	−0.33***	−0.11	− 0.11
Fear for the body after death	0.23	0.27**	**0.06**	0.27**	0.03	0.19*	**−0.06**	− 0.30**	0.11	− 0.08
Fear of premature death	**0.20**	0.46***	0.36*	0.41***	**0.25**^**+**^	0.40***	**−0.09**	− 0.49***	−0.10	− 0.14

Note: Volunteers: *N* = 42–50, paid employees: *N* = 115–128. + *p* < 0.10, * *p* < 0.05, ** *p* < 0.01, *** *p* < 0.001. Bold indicates cases where there is a significant difference in the strength of the relationship between the two groups

## Discussion

The aim of the present study was to compare the Hungarian hospice volunteers with paid employees with regard to attitudes regarding fear of death and mental health in order to shed light on their role in mental health and psychological well-being.

Hospice volunteers and employees consciously deal with the fear of death during their training, thus they are more aware of their own fears. That is why we found it pivotal to measure conscious fear of death. We believe that a deeper understanding of the aspects of fear of death will help raise awareness of these important issues, both in volunteer and in-service training, as they can be predictors of mental strain and quality of care.

Our results show that volunteers’ fear of death was lower than paid workers in most of the factors examined. The intensity and typical factors indicating stronger fear (such as *Fear of premature death and Fear for significant others*) were in line with earlier research findings and with international tendencies [3]. Previous studies [33, 34] have typically measured high levels of *Fear for significant others* and *the Fear of the dying process*, similar to the results of the current study.

For the past 20 years, Hungarian studies of thanatology and fear of death have been conducted among doctors, other healthcare workers, medical students and lay people [22, 35]. In accordance with our findings, these previous results have typically shown higher levels of fear of death among non-physician healthcare workers in the factors *Fear of dying process* and *Fear of premature death.*

Although both groups take part in hospice training, it is possible that volunteers’ previous experience with patients in critical condition may be more significant, therefore they deal with fear of death and death anxiety in a more conscious way. This may serve as explanation for the lower fear of death values. The mean age of our sample of Hungarian hospice volunteers is higher than that of their colleagues in Western Europe or in the United States [8, 36]. Conclusions must be drawn with caution because, as our results show, fear of death is higher among young people and the mean age of hospice workers was much lower than the volunteers’.

We also found that volunteers have higher psychological well-being and lower vital exhaustion than paid employees. Higher values of volunteers in the WHO Well-Being Questionnaire may confirm the hypothesis that the altruistic helping of others can affect general well-being [37, 38], because volunteers do not consider supporting patients to be “work”, but rather a kind of “service”. Among other things, this may be the cause of high vital exhaustion measured among hospice workers. The levels of perceived stress and depressive symptoms were practically the same for both examined groups. Claxton-Oldfield [39, 40] found low stress levels for volunteers. Similar perceived stress values in our sample are even more unexpected because based on our experience, we posited that paid employees would have lower perceived stress [41]. Yearly hospice statistics [42] show that hospice healthcare workers in Hungary are simultaneously working 2–3 jobs for living, so they are significantly overburdened. Unlike paid staff, the purpose of volunteering in hospice and palliative care is not to make money. In terms of old and new “styles” of volunteering, there is a mix in Hungary, in which solidarity plays an essential role, as well as spending one’s leisure time in a meaningful way and improving self-esteem. A 2008 study by Musick and Wilson reports similar experiences in American volunteers [5].

Regarding the correlates of fear of death, we found significant differences between the two groups. *Fear of the dying process* in both groups showed similar, moderate correlation with perceived stress, vital exhaustion and depression. This means that the more they are afraid of dying process, the higher their perceived stress, vital fatigue and depression. There was a similar, moderate relationship between fear of premature death and vital exhaustion in both groups, while the relationship between fear of premature death and perceived stress and depression was only significant among paid employees. Psychological well-being also showed a significant, moderate association in the five aspects of fear of death only among paid employees. In four of these (*Fear of the dying process, Fear of conscious death, Fear of premature death,* and *Fear of the body after death*), the relationship was found to be of moderate strength. Thus, various fears about death have a negative impact on the psychological well-being of paid workers in particular, but they are also associated with other aspects of mental health (depression, perceived stress, vital exhaustion) in both groups. As a result, this could be one of the intervention points, especially for paid workers.

According to our decades of training and everyday practical experience, these significant differences can be attributed to the fact that professionals establish a more direct, intimate, and continuous (physical and mental) relationship with patients during their care and handle their body after their death. They are confronted with the crude reality of illness and death, as opposed to the less medicalized involvement of volunteers.

As mentioned in the introduction, research for decades has focused on examining the physical and mental state of paid employees and their fear of death. We we intended to focus on increasing volunteering in Hungary in recent years [43]. Registered hospice volunteering, that is, working with a contract, is in a relatively early stage in Hungary. No national hospice volunteering database has been established in Hungary thus far. Hospice volunteers work in varying hours and jobs at the services: some work every day, some only on weekends, some are on call. Their jobs are diverse: gardening, legal counseling, assisting nurses in bathing and feeding patients, studying with children, baking bread, reading, praying, walking and shopping, etc. They organize fundraising and help in organizing events and shaping attitudes.

Our experience shows that hospice volunteers who work occasionally or regularly for one service are often not registered, and they do not participate in trainings either. This can also contribute to high staff turnover typical in this field. Claxton-Oldfield and colleagues scrutinized the issues of volunteer attrition and retention in their 2008 research project on a North-American population [44]. Pinpointing significant stressors for volunteers can improve stress relief and the recognition of common stressors. Working with terminally ill patients can be less stressful for volunteers because for them, this activity is different than their original profession [45].

The volunteers can experience stress in different ways, for instance if they feel they are not accepted by paid workers. It is important that we look at hospice care as a whole and not only interpret the situation of volunteers, but scrutinize it in context.

Compared to previous research, it was a welcome novelty that the proportion of volunteers among the respondents was high when examining hospice teams - approximately a quarter of the respondents. This could indicate a strengthening of the volunteer movement, an increase in their activity, and an opportunity to conduct further studies comparing volunteers and paid staff. Although volunteers do not get paid for working with patients, while professional staff does, the basic motivation in both groups is the desire to provide social support. According to the literature, altruistic attitudes are significant in the motivation of hospice workers [46].

Compared to the number of hospice volunteers working in other countries’, this figure is quite low in Hungary; our goal is to increase the number of skilled hospice volunteers and to help them stay in the profession, thereby enhancing the efficiency of the multidisciplinary team and the sustainable performance of paid staff.

### Limitations of the study

The main limitation of the investigation was that the cross-sectional method did not allow for drawing cause-effect conclusions. Furthermore, there was no information on people who refused to take part in the investigation, therefore there was no information on potential selection biases. Due to the low response rate and lack of male respondents, generalizability was substantially constrained. Additionally, we had no information on how many volunteers work bedside care.

The reverse items of MFODS decreased reliability, which could be due to the fact that the interpretation of reverse items might be more challenging, or that their meaning can be significantly different from the content of positive items; this question warrants further analysis.

Our result that volunteers in Hungary are on average 5 years older than the international/European mean is noteworthy. Differences in mean age should be taken into account because these may affect results, as older age may be associated with poorer health, bad mood, etc.

## Conclusion

Higher levels of psychological well-being and a lower fear of death among hospice volunteers indicate that they are less exhausted than paid employees. Therefore, an important conclusion of our research is that the results once again highlight the relationship between paid workers’ fear of death and their mental health and psychological well-being. Our assumption is that better acceptance of volunteers in hospices can help reduce the overload and exhaustion of paid employees, which may be the subject of further research. Volunteers are valuable assets, patients and their families appreciate the care and support provided by volunteers; they can make the work of other staff easier, provided staff are willing to rely on them. Our further goals are to establish a more efficient coordinating system, to form and maintain a community at the workplace, and thus enable hospice volunteers to become a full members of the team.

## Data Availability

The datasets used and/or analyzed during the current study are available from the corresponding author upon reasonable request.

## Abbreviations

HHPA: Hungarian Hospice-Palliative Association
MFODS: Multidimensional Fear of Death Scale
SD: Standard deviation
TUKEB: Medical Research Council, Tudományos és Kutatásetikai Bizottság

## Footnotes

1 In the original form of the questionnaire, the response is on a reverse scale (ie I totally agree with 1 point, and I strongly disagree with 5 points. This means that a lower score on the scales means more fear of death. However, in the current research, in the Hungarian version, we reversed the response and 1 point is low and 5 points is strong agreement. The reason for this was that we were afraid that the reverse thinking makes it difficult to complete the survey questionnaire and confuse the respondents. For this reason, unlike international studies, high scores indicate a higher level of fear of death in this study.

## References

[1] Block EM, Casarett DJ, Spence C, Gozalo P, Connor SR, Teno JM (2010). Got volunteers? Association of hospice use of volunteers with bereaved family members’ overall rating of the quality of end-of-life care. J Pain Symptom Manag.

[2] Hegedűs K, Szabó G, Zana Á (2008). Effect of end of life education on medical students’ and health care workers’ death attitude. Palliat Med.

[3] Zana Á, Szabó G, Hegedűs K (2009). Attitudes toward death in Hungary using the Multidimesional fear of death scale. Clinic and Experim Med J.

[4] Kegye A, Zana Á, Hegedűs K (2016). How does the suffering of cancer patients affect us? Reviewing the physical and mental well-being of hospice workers. Medycyna Paliatywna w Praktyce (Palliative Medicine in Practice).

[5] Musick MA, Wilson J (2008). Volunteers: a social profile.

[6] Stelzer EM, Lang FR (2016). Motivations of German hospice volunteers: how do they compare to nonhospice volunteers and US hospice volunteers?. Am J Hosp Palliat Care.

[7] Woitha K, Hasselaar J, van Beek K, Radbruch L, Jaspers B, Engels Y, Vissers K (2015). Volunteers in palliative care – a comparison of seven european countries: a descriptive study. Pain Pract.

[8] Schroeder S (2013). Rural Hospice in the United States: a Review of the Literature.

[9] Csikos A, Busa C, Muszbek K (2018). Hospice palliative care development in Hungary. J Pain Symptom Manag.

[10] Krakowiak P, Pawlovski L, Scott R, Howlett S (2018). Volunteering in hospice and palliative care in Poland and Eastern Europe. The changing face of volunteering in hospice and palliative care.

[11] Goossensen A, Somsen J, Scott R, Pelttari L (2016). Defining volunteering in hospice and palliative care in Europe: an EAPC white paper. Eur J Palliat Care.

[12] Whitebird RR, Asche SE, Thompson GL, Rossom R, Heinrich R (2013). Stress, burnout, compassion fatigue, and mental health in hospice workers in Minnesota. J Palliat Med.

[13] Barnett MD, Ruiz IA (2018). Psychological distress and compassion fatigue among hospice nurses: the mediating role of self-esteem and negative affect. J Palliat Med.

[14] Amenta MM (1984). Death anxiety, purpose in life and duration of service in hospice volunteers. Psychol Rep.

[15] Békés V (2000). Ki fél a haláltól? [Who is afraid of death?] Kharón.

[16] Lehto RH, Stein KF (2009). Death anxiety: an analysis of an evolving concept. Res Theory Nurs Pract.

[17] Neimeyer RA (1994). Death anxiety handbook: research, instrumentation, and application.

[18] Robbins RA, Neimeyer RA (1994). Death competency: Bugen’s coping with death scale and death self-efficacy. Death anxiety handbook: research, instrumentation, and application.

[19] Garbay M, Gay MC, Claxton-Oldfield S (2015). Motivations, death anxiety, and empathy in hospice volunteers in France. Am J Hosp Palliat Care.

[20] Scherwitz L, Pullman M, McHenry P, Gao B, Ostaseski F (2006). A contemplative care approach to training and supporting hospice volunteers: a prospective study of spiritual practice, well-being, and fear of death. Explore (NY).

[21] Claxton-Oldfield S, Crain M, Claxton-Oldfield J (2007). Death anxiety and death competency: the impact of a palliative care volunteer training program. Am J Hosp Palliat Care.

[22] Zana Á, Konkolÿ Thege B, Limpár I, Henczi E, Golovics P, Pilling J, Hegedűs K (2014). Összefüggésbe hozható-e a halálfélelem a foglalkozással? [Is profession associated with fear of death?]. Orv Hetil.

[23] Neimeyer RA, Moore MK, Neimeyer RA (1994). Validity and reliability of the multidimensional fear of death scale. Death anxiety handbook: research, instrumentation, and application.

[24] Zana Á, Hegedűs K, Szabó G (2006). A Neimeyer és Moore-féle Multidimenzionális Halálfélelem Skála validálása magyar populáción [Validity and reliability of Multidimensional Fear of Death Scale in Hungarian population]. Mentálhigiéné és Pszichoszomatika.

[25] Cohen S, Tyrrell DA, Smith AP (1993). Negative life events, perceived stress, negative affect, and susceptibility to the common cold. J Pers Soc Psychol.

[26] Stauder A, Konkolӱ TB (2006). Az Észlelt Stressz Kérdőív (PSS) Magyar verziójának jellemzői [Characteristics of the Hungarian version of the Perceived Stress Scale (PSS)]. Mentálhigiéné és Pszichoszomatika.

[27] Bech P, Gudex C, StaehrJohansen K (1996). The WHO (ten) well-being index: validation in diabetes. Psychother Psychosom.

[28] Susánszky É, Konkoly Thege B, Stauder A, Kopp M (2006). A WHO Jól-lét kérdőív rövidített (WBI-5) magyar változatának validálása a Hungarostudy 2002 országos lakossági egészségfelmérés alapján [Validation of the short (5-item) version of the WHO Well-Being Scale based on a Hungarian representative health survey (Hungarostudy 2002)]. Mentálhigiéné és Pszichoszomatika.

[29] Kopp M, Rózsa S, Skrabski Á, Kopp M, Kovács ME (2008). A Hungarostudy 2002 és a Hungarostudy 2006 követéses vizsgálat kérdőívei [The questionnaires of the 2002 and the 2006 follow-up Hungarostudy]. A magyar népesség életminősége az ezredfordulón [The quality of life of the Hungarian population at the turn of the millenium].

[30] Susánszky É, Székely A, Susánszky É, Szántó ZS (2013). A Hungarostudy 2013 felmérés módszertana [The methodology of Hungarostudy 2013]. Magyar lelkiállapot 2013 [Hungarian State of Mind 2013].

[31] Kopp MS, Falger PR, Appels A, Szedmák S (1998). Depressive symptomatology and vital exhaustion are differentially related to behavioral risk factors for coronary artery disease. Psychosom Med.

[32] Cohen J (1988). Statistical power analysis for the behavioral sciences.

[33] Cicirelli VG (2001). Personal meanings of death in older adults and young adults in relation to their fears of death. Death Stud.

[34] Neimeyer RA, Wittkowski J, Moser RP (2004). Psychological research on death attitudes: an overview and evaluation. Death Stud.

[35] Zana Á, Szabó G, Hegedűs K (2009). The development and change of death image in Hungary. Differences in value judgement according to age and analysis of possible measurement methods. Is death still a taboo?. Thesis Clin Exp Med J.

[36] Morris SM, Payne S, Ockenden N, Hill M (2017). Hospice volunteers: bridging the gap to the community?. Health Soc Care Community.

[37] Thoits PA, Hewitt LN (2001). Volunteer work and well-being. J Health Soc Behav.

[38] Schwartz C, Meisenhelder JB, Ma Y, Reed G (2003). Altruistic social interest behaviors are associated with better mental health. Psychosom Med.

[39] Claxton-Oldfield S (2016). Hospice palliative care volunteers: a review of commonly encountered stressors, how they cope with them, and implications for volunteer training/management. Am J Hosp Palliat Care.

[40] Claxton-Oldfield S, Scott R, Howlett S (2018). Volunteering in hospice palliative care in Canada. The changing face of volunteering in hospice and palliative care.

[41] Pavelková H, Bužgová R (2015). Burnout among healthcare workers in hospice care. Central Eur J of Nurse Midwifery.

[42] Hegedűs K, Munk K (2018). Hospice Magyarországon 2017. Statisztikai áttekintés. Hospice in Hungary. Statistical review. Kharón.

[43] Zana A, Czegledi E, Kegye A, Hegedus K (2018). Differences in Well-being and Fear of Death of Hospice Employees and Volunteers in Hungary. Palliat Med.

[44] Claxton-Oldfield S, Claxton-Oldfield J (2008). Keeping hospice palliative care volunteers on board: dealing with issues of volunteer attrition, stress, and retention. Indian J Palliat Care.

[45] Khvorostianov N, Remennick L (2017). ‘By helping others, we helped ourselves’: Volunteering and Social Integration of Ex-Soviet Immigrants in Israel. Voluntas.

[46] Cabin WD. Actualizing “professional altruism”: a comparison of home health care and hospice social workers. Home Health Care Manag Pract. 2008;20:474–81.

